# Posterior parietal cortex mediates fear renewal in a novel context

**DOI:** 10.1186/s13041-020-0556-y

**Published:** 2020-02-05

**Authors:** Bitna Joo, Ja Wook Koo, Sukwon Lee

**Affiliations:** 1 Korea Brain Research Institute (KBRI), 61 Cheomdan-ro, Dong-gu, Daegu, 41068 Republic of Korea; 2 Department of Brain and Cognitive Sciences, Daegu Gyeongbuk Institute of Science and Technology (DGIST), 333 Techno Jungang-daero, Hyeonpung-myeon, Dalseong-gun, Daegu, 42988 Republic of Korea

**Keywords:** Posterior parietal cortex, Learning and memory, Fear renewal, Optogenetics, Novel context

## Abstract

The return of fear following extinction therapy is an important issue associated with the treatment of many fear-related disorders. Fear renewal is a suitable model, with which context-dependent modulation of the fear response can be examined. In this model, any context outside of an extinction context (e.g., novel or familiar contexts) could evoke relapse of the fear response. However, brain regions associated with context-dependent modulation are not fully understood. The posterior parietal cortex (PPC) is considered a center for integrating multisensory information and making decisions. To study its role in the contextual modulation of fear relapse, we reversibly inactivated the PPC in mice before they were exposed to various contexts after extinction training. When muscimol was infused into the PPC, fear renewal was impaired in a novel context, but not in a familiar context. Fear relapses were blocked during optogenetic inhibition of the PPC, only when animals were placed in a novel context. We propose that the neural activity of the PPC is necessary for the relapse of a precise response to an extinguished conditioned stimulus in a novel context.

## Introduction

In a natural environment, animals encounter various contextual situations and can perceive whether they may face imminent danger. Sometimes, the same stimulus can have distinct meanings depending on the contextual situation. Evaluation of the context to which the animal is exposed is essential for dealing with a dynamic environment and increasing the probability of survival. However, the specific brain regions that mediate context-dependent modulation of the fear response have not been fully investigated. To understand this phenomenon, the fear renewal model has been widely used. In Pavlovian fear conditioning, a conditioned stimulus (CS; e.g. sound) is paired with an unconditioned stimulus (US; e.g. an electric foot shock) to produce a fear response (e.g. freezing behavior). A strong fear response is weakened by repetitive presentations of the CS without the US (extinction) [1–3]. Many researchers have demonstrated that the context or external environment in which an animal is exposed to stimuli can have an effect on the learned fear response to CS. After extinction learning, the contextual effect becomes important to evoke a fear response, and an extinguished fear response can easily return outside of the extinction context (fear renewal) [3–6].

Different types of context have been known to evoke fear renewal, including the novel context, in which animals undergo neither fear conditioning nor extinction [1, 7, 8], and the familiar context, in which animals are exposed to fear conditioning [9, 10]. The amygdala and hippocampus mediate fear renewal in both types of context [11–13]. During fear renewal, synaptic efficacy of the thalamo-amygdala pathway increases. Some synaptic mechanisms for this increase in synaptic efficacy have been studied: the activity of GluA2-lacking the AMPA receptor is increased [14], and the phosphorylation of GluA1 at serine 831 is required to produce fear renewal [15].

Pharmacological and electrolytic lesions of the dorsal or ventral hippocampus block fear renewal in both types of context [16–19]. However, neural circuits or substrates that may be differentially regulated by various types of context have remained generally unidentified. Disconnection between the ventral hippocampal and prefrontal projections impairs fear renewal in a novel context, but not in extinction retrieval [11]. Furthermore, kappa opioid receptors in the ventral hippocampus mediate renewal in a familiar context, but not in a novel context [20, 21]. These data raise questions regarding the specific brain region responsible for processing novel contextual information.

The posterior parietal cortex (PPC) receives diverse sensory and cognitive inputs [22–25] and integrates multisensory signals [26–28]. Sensory information is processed by the PPC and transformed into behavioral activity. The response of PPC neurons affects cognitive processes [29, 30]. Lesion studies have revealed that the PPC mediates many context-dependent tasks such as object recognition [31–35]. Moreover, PPC neurons that are activated during contextual fear conditioning, are reactivated when the fear memory is retrieved [36]. These findings support the idea that the PPC is implicated in the storage of memory or processing of sensory information acquired from the external environment.

In the present study, we investigated the role of the PPC in fear renewal paradigms with novel and familiar contexts. Inactivation of the PPC selectively blocked fear renewal in a novel context, but not in a familiar context. Our results demonstrated that the PPC differentially modulates the retrieval of conditioned fear after extinction, depending on the types of context.

## Results

### Inactivation of the PPC attenuates renewal of extinguished fear in a novel context (ABC renewal)

**Fig. 1:**
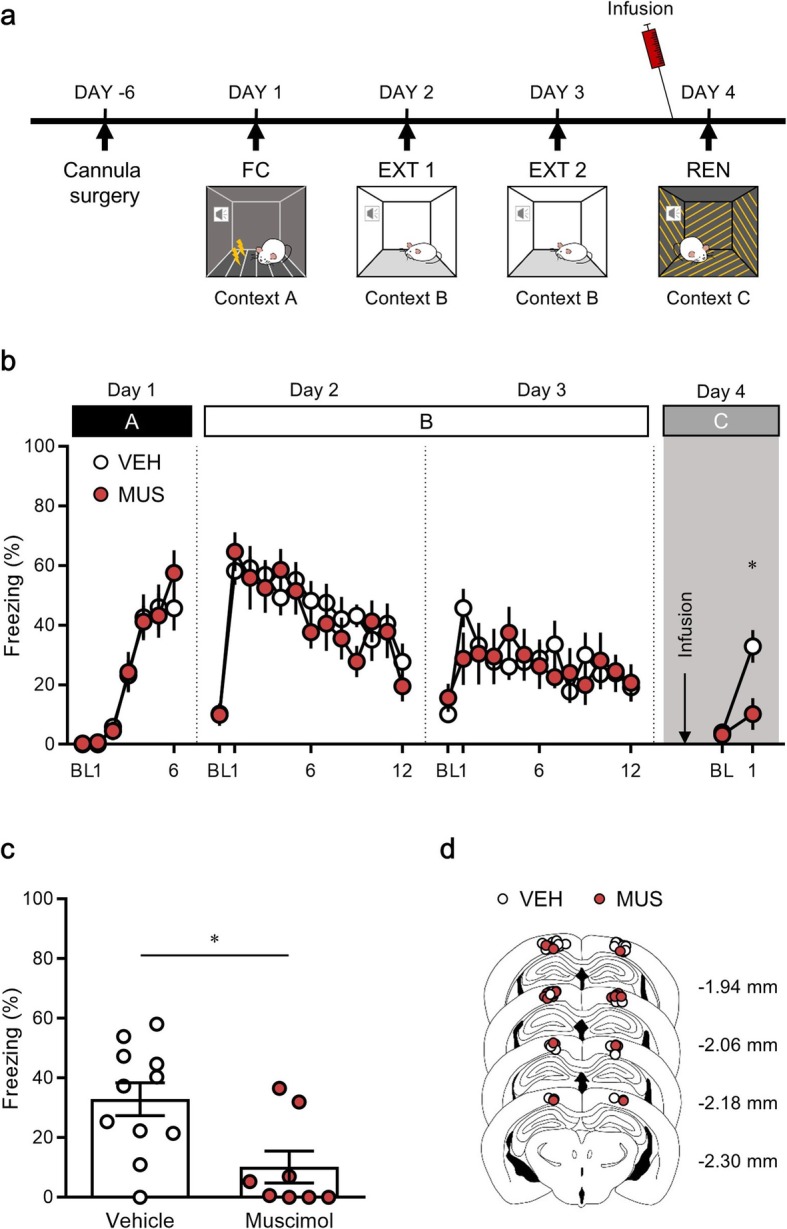
Pharmacological inactivation of the posterior parietal cortex (PPC) prevents fear renewal in a novel context. **a**, Schematic diagram showing the experimental procedure for fear renewal with drug infusion. **b**, Percentage of freezing behavior across the conditioning, extinction and renewal sessions. Each dot represents the level of freezing when the conditioned stimulus (CS) was presented, except the first dot of each session, which shows the pre-CS baseline. **c**, Inactivation of the PPC significantly attenuated fear renewal in a novel context. **d**, Illustration of injector cannula tips in the PPC. FC, fear conditioning; EXT, extinction; REN, renewal; BL, baseline; VEH, vehicle; MUS, muscimol. *P < 0.05

To explore the role of the PPC in differential modulation of fear memory depending on the various types of context, we first determined the effect of PPC inactivation on fear renewal in a novel context, in which neither fear conditioning nor extinction had occurred. Thus, mice were placed in an environment they had never encountered before. Mice were first exposed to pairings of the CS and US in context A (fear conditioning). They were then placed in another context (context B) and received the CS without the US over the next 2 days (extinction). On the day of fear renewal testing, mice were randomly assigned to each experimental group and either muscimol, a gamma-aminobutyric acid (GABA) receptor type A agonist, or a vehicle was microinfused into the PPC 10 min before the test. Mice were placed in the novel context (context C), and a single CS was then presented for the test of fear renewal. During the 5-min test, fear responses were measured based on the duration of freezing behavior (Fig. 1a).

Mice showed no significant differences in freezing behavior between the vehicle and muscimol groups during: fear conditioning on day 1 [time × drug treatment interaction, *F* (5, 85) = 0.50, *P* = 0.78; time, *F* (5, 85) = 32.21, *P* < 0.0001; drug treatment, *F* (1, 17) = 0.07, *P* = 0.79, two-way repeated measures analysis of variance (ANOVA) (Fig. 1b)]; the first extinction session on day 2 [time × drug treatment interaction, *F* (11, 187) = 1.27, *P* = 0.24; time, *F* (11, 187) = 11.53, *P* < 0.0001; drug treatment, *F* (1, 17) = 0.21, *P* = 0.65, two-way repeated measures ANOVA (Fig. 1b)]; and the second extinction session on day 3 [time × drug treatment interaction, *F* (11, 187) = 1.56, *P* = 0.11; time, *F* (11, 187) = 2.28, *P* = 0.01; drug treatment, *F* (1, 17) = 0.03, *P* = 0.87, two-way repeated measures ANOVA (Fig. 1b)].

On day 4, the test for fear relapse was performed. After inactivation of the PPC, significant fear renewal was examined relative to the last CS of extinction in the vehicle group (extinction, 19.22 ± 4.90, renewal, 32.89 ± 5.53, *n* = 11, *P* = 0.03, paired *t-*test), but not in the muscimol group (extinction, 20.67 ± 6.23, renewal, 10.18 ± 5.36, *n* = 8, *P* = 0.09, paired *t-*test; Fig. 1b). The muscimol group showed significantly diminished levels of freezing behavior compared with the vehicle group (vehicle group, 32.89 ± 5.53, n = 11; muscimol group, 10.18 ± 5.36, n = 8, *P* = 0.01, unpaired *t-*test; Fig. 1b, c). After the test of fear renewal, the placement of the cannula tip was verified (Fig. 1d). These results revealed that inhibition of the PPC attenuated fear renewal in a novel context.

### Inactivation of the PPC does not disrupt fear renewal in a familiar context (ABA renewal)

**Fig. 2:**
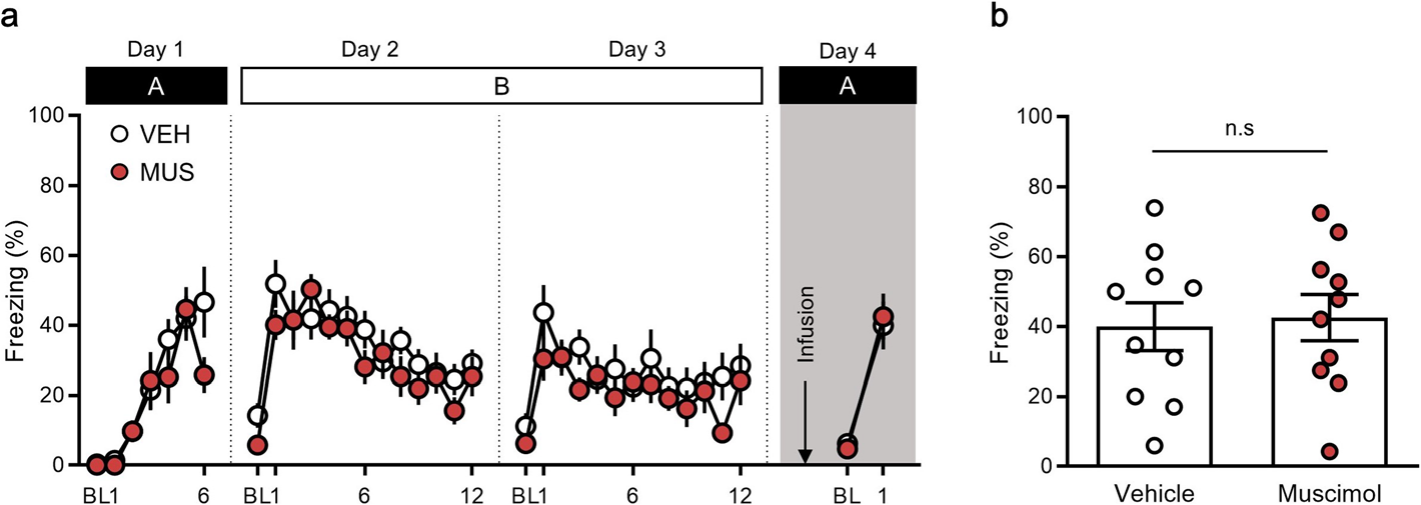
Pharmacological inactivation of the posterior parietal cortex (PPC) does not affect fear renewal in a familiar context. **a,** Percentage of freezing behavior across the conditioning, extinction and renewal sessions. Each dot represents the level of freezing when the conditioned stimulus (CS) was presented, except the first dot of each session, which shows the pre-CS baseline. **b**, The PPC was not required for fear renewal in a familiar context, in which mice were exposed to fear conditioning. BL, baseline; VEH, vehicle; MUS, muscimol

Renewal occurs both in a novel context and a familiar context, in which mice are exposed to fear conditioning [1, 17, 37]. We evaluated whether inactivation of the PPC might influence fear renewal in a familiar context. Fear responses to the CS showed no significant differences between the vehicle and muscimol groups during: fear conditioning on day 1 [time × drug treatment interaction, *F* (5, 90) = 1.64, *P* = 0.16; time, *F* (5, 90) = 19.62, *P* < 0.0001; drug treatment, *F* (1, 18) = 0.83, *P* = 0.37, two-way repeated measures ANOVA (Fig. 2a)]; the first extinction session on day 2 [time × drug treatment interaction, *F* (11, 198) = 0.97, *P* = 0.47; time, *F* (11, 198) = 8.37, *P* < 0.0001; drug treatment, *F* (1, 18) = 0.75, *P* = 0.40, two-way repeated measures ANOVA (Fig. 2a)]; and second extinction session on day 3 [time × drug treatment interaction, *F* (11, 198) = 1.03, *P* = 0.42; time, *F* (11, 198) = 3.52, *P* = 0.0002; drug treatment, *F* (1, 18) = 1.04, *P* = 0.32, two-way repeated measures ANOVA (Fig. 2a)].

On the following day, after microinjection of muscimol or the vehicle, the CS was presented in the familiar context in which fear conditioning occurred (context A; Fig. 2a). Significant fear renewal was observed relative to the last CS of extinction both in the vehicle (extinction, 28.41 ± 6.26, renewal 39.94 ± 6.85, *n* = 10, *P* = 0.04, paired *t-*test) and muscimol group (extinction, 24.14 ± 6.98, renewal, 42.50 ± 6.66, n = 10, *P* = 0.04, paired *t-*test; Fig. 2a). Interestingly, in contrast to the results of fear renewal in the novel context (Fig. 1), no statistically significant differences were noted between the muscimol and vehicle groups (vehicle group, 39.94 ± 6.85, *n* = 10; muscimol group, 42.50 ± 6.66, n = 10, *P* = 0.93, unpaired *t-*test; Fig. 2a, b). These results indicate that inhibition of the PPC did not affect fear renewal in the familiar context.

### The PPC is not required for fear expression in the extinction context (extinction retrieval)

**Fig. 3:**
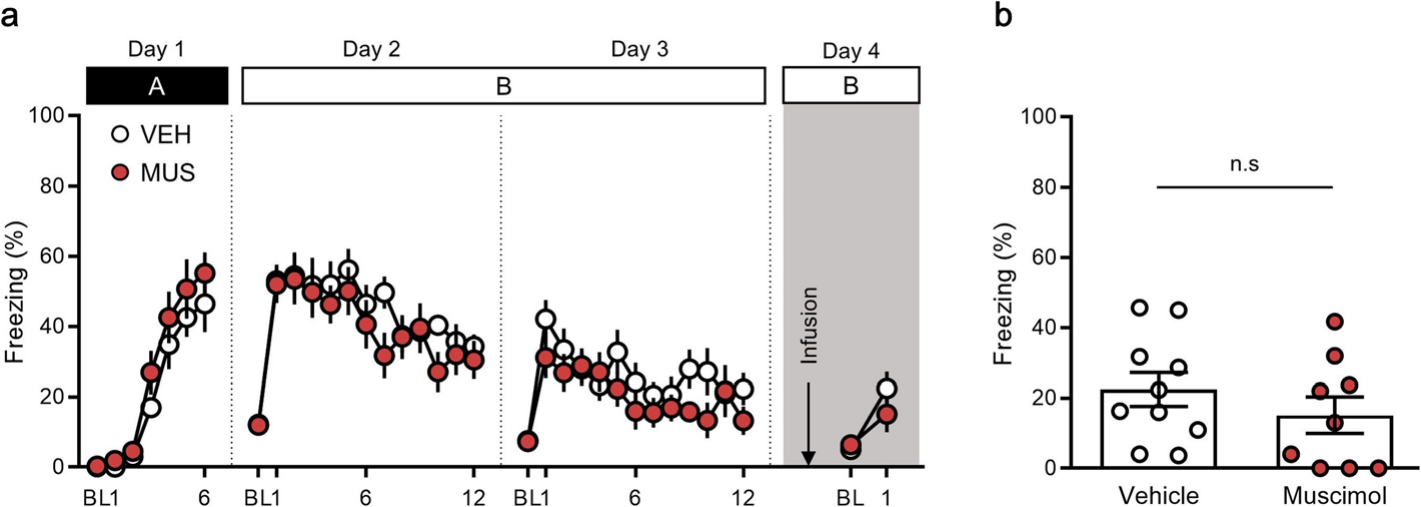
Pharmacological inactivation of the posterior parietal cortex (PPC) does not block extinction retrieval. **a,** Percentage of freezing behavior across the conditioning, extinction and extinction retrieval sessions. Each dot represents the level of freezing when the conditioned stimulus (CS) was presented, except the first dot of each session, which shows the pre-CS baseline. **b**, Inactivation of the PPC has no effect on extinction retrieval in a familiar context. BL, baseline; VEH, vehicle; MUS, muscimol

To better understand the function of the PPC depending on the contextual situation, we next evaluated the fear response in the extinction context, in which mice experienced the CS without the US after extinction training was performed. No significant differences in freezing behaviors were detected between the vehicle and muscimol groups during: fear conditioning on day 1 [time × drug treatment interaction, *F* (5, 85) = 0.28, *P* = 0.92; time, *F* (5, 85) = 39.44, *P* < 0.0001; drug treatment, *F* (1, 17) = 1.91, *P* = 0.18, two-way repeated measures ANOVA (Fig. 3a); first extinction session on day 2 [time × drug treatment interaction, *F* (11, 187) = 0.84, *P* = 0.60; time, *F* (11, 187) = 7.40, *P* < 0.0001; drug treatment, *F* (1, 17) = 0.72, *P* = 0.41, two-way repeated measures ANOVA (Fig. 3a)]; and second extinction session on day 3 [time × drug treatment interaction, *F* (11, 187) = 0.97, *P* = 0.47; time, *F* (11, 187) = 4.10, *P* < 0.0001; drug treatment, *F* (1, 17) = 1.53, *P* = 0.23, two-way repeated measures ANOVA (Fig. 3a)].

On day 4, mice were placed in the extinction context (context B) and examined the levels of freezing behavior after drug administration (Fig. 3a). Extinction retrieval was investigated relative to the last CS of extinction both in the vehicle (extinction, 22.27 ± 4.62, retrieval, 22.43 ± 4.80, *n* = 10, *P* = 0.49, paired *t-*test) and muscimol group (extinction, 13.25 ± 4.10, retrieval, 15.15 ± 5.17, *n* = 9, *P* = 0.38, paired *t-*test; Fig. 3a). We observed no significant differences between the muscimol and vehicle groups (vehicle group, 22.43 ± 4.80, n = 10; muscimol group, 15.15 ± 5.17, n = 9, *P* = 0.32, unpaired *t-*test; Fig. 3a, b). These results demonstrate that pharmacological inactivation of the PPC has no effect on the expression of fear in response to a CS in the extinction context.

### PPC inactivation does not block fear reinstatement in the extinction context

**Fig. 4:**
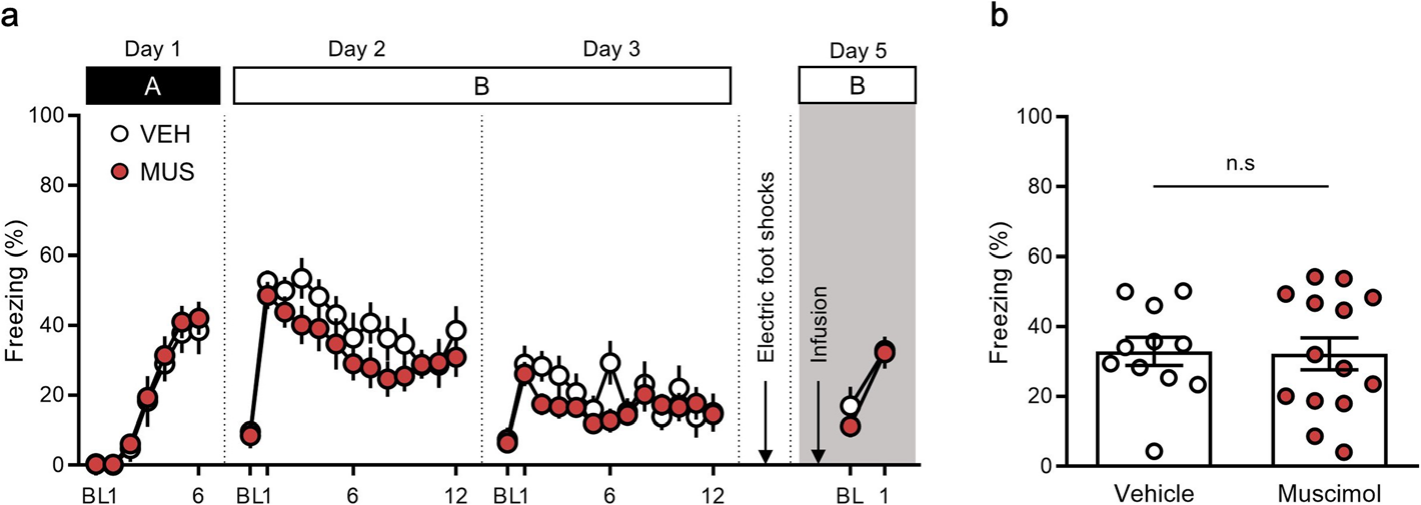
Pharmacological inactivation of the posterior parietal cortex (PPC) does not impair fear reinstatement. **a**, Percentage of freezing behavior across the conditioning, extinction and reinstatement sessions. Each dot represents the level of freezing when the conditioned stimulus (CS) was presented, except the first dot of each session, which shows the pre-CS baseline. **b**, Inactivation of the PPC does not block fear reinstatement in a familiar context. BL, baseline; VEH, vehicle; MUS, muscimol

Fear reinstatement is another type of fear relapse that may occur after extinction. We evaluated whether inactivation of the PPC might impair fear reinstatement. Fear reinstatement occurs when the aversive US is again presented after extinction [4, 37, 38]. No statistical differences were observed in the fear response between the vehicle and muscimol groups during: fear conditioning on day 1 [time × drug treatment interaction, *F* (5, 115) = 0.06, *P* = 0.99; time, *F* (5, 115) = 44.71, *P* < 0.0001; drug treatment, *F* (1, 23) = 0.17, *P* = 0.68, two-way repeated measures ANOVA (Fig. 4a)]; first extinction session on day 2 [time × drug treatment interaction, *F* (11, 253) = 0.64, *P* = 0.79; time, *F* (11, 253) = 7.62, *P* < 0.0001; drug treatment, *F* (1, 23) = 1.56, *P* = 0.22, two-way repeated measures ANOVA (Fig. 4a)]; and second extinction session on day 3 [time × drug treatment interaction, *F* (11, 253) = 1.58, *P* = 0.11; time, *F* (11, 253) = 3.19, *P* = 0.0005; drug treatment, *F* (1, 23) = 0.90, *P* = 0.35, two-way repeated measures ANOVA (Fig. 4a)]. On the following day, mice received an unsignalled foot shock twice, to reinstate the fear response. One day after reinstatement of the shock, either muscimol or the vehicle was injected into the PPC of mice, and the levels of freezing behavior during presentation of the CS were measured (context B). Fear reinstatement was confirmed relative to the last CS of extinction both in the vehicle (extinction, 15.07 ± 5.43, reinstatement, 32.85 ± 4.02, *n* = 11, *P* = 0.005, paired *t-*test) and muscimol group (extinction, 14.55 ± 3.52, reinstatement, 32.13 ± 4.59, *n* = 14, *P* = 0.005, paired *t-*test; Fig. 4a). No significant differences were noted between the muscimol and vehicle groups (vehicle group, 32.85 ± 4.02, n = 11; muscimol group, 32.13 ± 4.59, n = 14, *P* = 0.91, unpaired *t-*test; Fig. 4a, b). These results indicate that the PPC is not required for fear reinstatement.

### The activity of the PPC is not necessary for retrieval of fear memory in a novel context

**Fig. 5:**
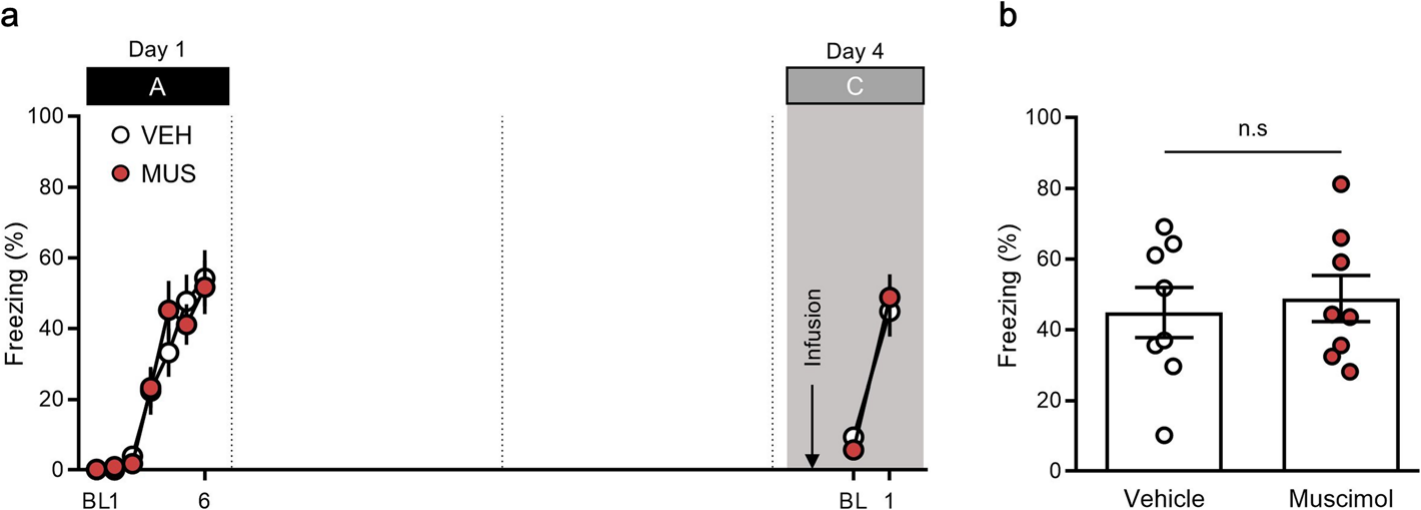
Pharmacological inactivation of the posterior parietal cortex (PPC) does not prevent retrieval of fear memory in a novel context. **a,** Percentage of freezing behavior across the conditioning and retrieval sessions. Each dot represents the level of freezing when the conditioned stimulus (CS) was presented, except the first dot of each session, which shows the pre-CS baseline. **b**, Inactivation of the PPC has no effect on retrieval of conditioned fear memory in a novel context. BL, baseline; VEH, vehicle; MUS, muscimol

During fear renewal in a novel context, the effect of inactivation of the PPC may be due to the impairment of freezing ability in context C or impairment in the retrieval of fear memory. To verify this theory, we determined the effects of muscimol administration in the PPC on fear retrieval in a novel context without extinction training. Freezing behaviors showed no significant differences between the vehicle and muscimol groups during: fear conditioning [time × drug treatment interaction, *F* (5, 70) = 0.76, *P* = 0.58; time, *F* (5, 70) = 36.61, *P* < 0.0001; drug treatment, *F* (1, 14) = 0.007, *P* = 0.93, two-way repeated measures ANOVA (Fig. 5a)]. Two days after fear conditioning, the fear retrieval test was conducted in a novel context (context C). The muscimol and vehicle groups showed no significant differences in freezing behavior (vehicle group, 44.83 ± 7.15, *n* = 8; muscimol group, 48.78 ± 6.52, n = 8, *P* = 0.69, unpaired *t-*test; Fig. 5a, b). Thus, these results demonstrate that inactivation of the PPC does not affect retrieval of the conditioned fear memory in a novel context.

### Optogenetic inhibition of the PPC activity disrupts fear renewal in a novel context (ABC renewal)

**Fig. 6:**
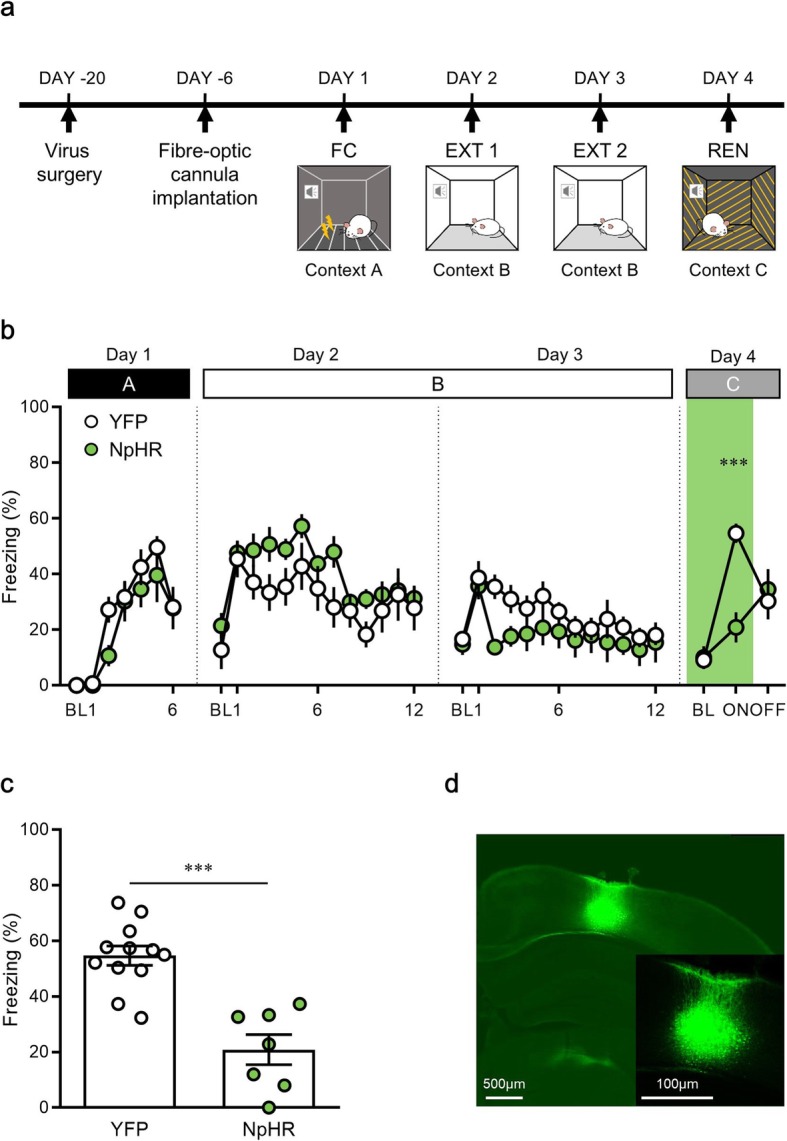
Optogenetic inhibition of the posterior parietal cortex (PPC) attenuates fear renewal in a novel context. **a**, Schematic diagram showing the experimental procedure for fear renewal with optogenetic manipulation. **b**, Percentage of freezing behavior across the conditioning, extinction and renewal sessions. No significant changes were noted in fear responses to the conditioned stimulus (CS) during fear conditioning and extinction before optogenetic manipulation. On the day of fear renewal, fear response is significantly blocked during a light-on trial, but not a light-off trial. Each dot represents the level of freezing when the CS was presented, except the first dot of each session, which shows the pre-CS baseline. **c,** Photo-inactivation of the PPC significantly impairs fear renewal in a novel context. **d**, Representative image of viral expression in the PPC. FC, fear conditioning; EXT, extinction; REN, renewal; BL, baseline; ON, light on trial; OFF, light off trial; ***P < 0.0001

To achieve better temporal resolution in the fear renewal experiment, we adopted an optogenetic inhibition technique. An adeno-associated virus (AAV) carrying halorhodopsin (NpHR), which hyperpolarizes the neuronal membrane under light stimulation, was injected into the PPC 3 weeks before behavioral training started. After extinction training, the mice were exposed to a novel context (context C), in which neither fear conditioning (context A) nor extinction (context B) had occurred (Fig. 6a). No significant differences were observed in freezing behavior between yellow fluorescent protein (YFP) and NpHR groups during: fear conditioning on day 1 [time × group interaction, *F* (5, 85) = 0.91, *P* = 0.48; time, *F* (5, 85) = 21, *P* < 0.0001; group, *F* (1, 17) = 1.6, *P* = 0.23, two-way repeated measures ANOVA (Fig. 6b)]; the first extinction session on day 2 [time × group interaction, *F* (11, 187) = 0.93, *P* = 0.51; time, *F* (11, 187) = 6.0, *P* < 0.0001; group, *F* (1, 17) = 3.19, *P* = 0.09, two-way repeated measures ANOVA (Fig. 6b)]; and second extinction session on day 3 [time × group interaction, *F* (11, 187) = 0.84, *P* = 0.60; time, *F* (11, 187) = 3.73, *P* < 0.0001; group, *F* (1, 17) = 2.77, *P* = 0.11, two-way repeated measures ANOVA (Fig. 6b)].

On day 4, the relapse of fear was tested in the novel context (context C). Mice displayed significant fear renewal relative to the last CS of extinction in the YFP group while optogenetic inactivation (extinction, 18.08 ± 3.60, renewal, 54.66 ± 3.44, *n* = 12, *P* < 0.0001, paired *t*-test), but not in the NpHR group (extinction, 15.46 ± 7.21, renewal, 20.87 ± 5.45, *n* = 7, *P* = 0.26, paired *t*-test; Fig. 6b). The NpHR group showed a significant difference in freezing levels induced by photo-inactivation compared with the YFP group in the (light-on) ON session (YFP group, 54.66 ± 3.44, *n* = 12; NpHR group, 20.87 ± 5.45, *n* = 7, *P* < 0.001, unpaired *t*-test; Fig. 6b, c), but not in (light-off) OFF session (YFP group, 30.28 ± 6.50, *n* = 12; NpHR group, 34.49 ± 7.26, *n* = 7, *P* = 0.68, unpaired *t*-test; Fig. 6b). Furthermore, during a 5-min exploration, mice showed a minimal level of contextual freezing before the presentations of CSs (YFP group, 9.11 ± 3.25, *n* = 12; NpHR group, 10.33 ± 4.06, *n* = 7, *P* = 0.86, unpaired *t-*test; Fig. 6c). After the behavioral test, viral expression in the PPC was verified (Fig. 6d). These results reveal that inhibition of the PPC attenuates fear renewal in a novel context.

### Optogenetic inactivation of the PPC does not impair fear renewal in a familiar context (ABA renewal)

**Fig. 7:**
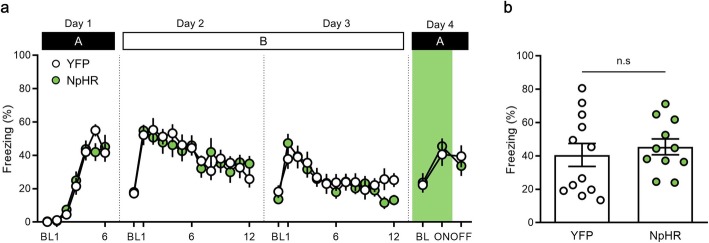
Optogenetic inactivation of the posterior parietal cortex (PPC) does not block fear renewal in a familiar context. **a,** Percentage of freezing behavior across the conditioning, extinction and renewal sessions. Each dot represents the level of freezing when the CS was presented, except the first dot of each session, which shows the pre-CS baseline. **b**, Inactivation of the PPC has no effect on renewal of extinguished fear memory in a familiar context. BL, baseline; ON, light-on trial; OFF, light-off trial

Renewal occurs both in a novel context and a familiar context, in which mice are exposed to fear conditioning [1, 17, 37]. We evaluated whether inactivation of the PPC might influence fear renewal in a familiar context. After extinction training, mice were exposed to a familiar context (context A), in which they had experienced fear conditioning. Mice showed no significant change in freezing behavior between the YFP and NpHR groups during: fear conditioning on day 1 [time × group interaction, *F* (5, 105) = 1.26, *P* = 0.29; time, *F* (5, 105) = 51.36, *P* < 0.0001; group, *F* (1, 21) = 0.006, *P* = 0.94, two-way repeated measures ANOVA (Fig. 7a)]; the first extinction session on day 2 [time × group interaction, *F* (11, 231) = 0.90, *P* = 0.54; time, *F* (11, 231) = 7.25, *P* < 0.0001; group, *F* (1, 21) = 0.003, *P* = 0.95, two-way repeated measures ANOVA (Fig. 7a)]; and second extinction session on day 3 [time × group interaction, *F* (11, 231) = 1.67, *P* = 0.08; time, *F* (11, 231) = 10.47, *P* < 0.0001; group, *F* (1, 21) = 0.20, *P* = 0.66, two-way repeated measures ANOVA (Fig. 7a)].

On day 4, fear renewal was investigated relative to the last CS of extinction both in the YFP (extinction, 25.22 ± 4.22, renewal, 40.55 ± 6.90, *n* = 12, *P* = 0.03, paired *t-*test) and NpHR group (extinction, 13.17 ± 3.18, renewal, 45.43 ± 4.73, *n* = 11, *P* < 0.0001, paired *t-*test; Fig. 7a). optogenetic inhibition was performed during baseline (BL) and the presentation of the first CS (ON), and then lights were turned off during the presentation of the second CS (OFF) (Fig. 7a). In a familiar context (context A), the levels of freezing behavior showed no significant differences between the NpHR group and the YFP group during optogenetic inactivation (YFP group, 40.55 ± 6.90, *n* = 12; NpHR group, 45.43 ± 4.73, *n* = 11, *P* = 0.57, unpaired *t-*test; Fig. 7b). Furthermore, no significant differences in freezing behavior were observed in the OFF session (YFP group, 39.38 ± 6.67, *n* = 12; NpHR group, 33.68 ± 6.02, *n* = 11, *P* = 0.53, unpaired *t-*test; Fig. 7a). Consistent with the pharmacological inactivation (Fig. 2), these results demonstrate that optogenetic inhibition of the PPC has no effect on fear renewal in a familiar context.

## Discussion

Our findings suggest a novel role of the PPC in fear renewal. We demonstrated that optogenetic and pharmacological inactivation of the PPC selectively disrupts fear renewal in a novel context, in which neither fear conditioning nor extinction has occurred (ABC renewal). In contrast, silencing the activity of the PPC did not affect fear renewal in a familiar context, in which fear conditioning was performed (ABA renewal). In the extinction context (context B), retrieval of the extinguished fear memory and fear reinstatement were not impaired by PPC inactivation. Moreover, inhibition of the PPC did not prevent the retrieval of fear memory in a novel context.

A previous report suggests that lesions of the PPC does not disrupt fear conditioning, nor extinction of auditory and contextual fear memory [39]. These findings support the idea that the effect of silencing PPC activity on fear renewal in a novel context is neither caused by any defect in the retrieval of fear memory nor the impairment of freezing ability in context C. Overall, our findings suggest a unique role of the PPC that is necessary to produce the renewal of an extinguished fear memory in a novel context, but not in a familiar context.

The important issue that remains is why inactivation of the PPC impairs fear renewal in a novel context alone. One possible explanation is that renewal of an extinguished fear memory in the novel context requires PPC activity caused by contextual novelty. The PPC might play an essential role in the processing of contextual novelty, along with other previously established novelty-activated brain regions, such as the hippocampus and ventral tegmental area [40–42]. Contextual novelty itself may act as a signal to evoke a behavioral response. To determine contextual novelty, the associative cortex might be applicable, because unlike unimodal signals, such as a CS, contextual stimuli are usually persistent multisensory signals that include visual, auditory, olfactory and tactile stimuli [4, 5]. The PPC reportedly acts as a convergence center for multimodal signals [22, 24]. We propose that the PPC could serve as a potential area, in which the processing of contextual novelty may occur. Our results support the idea that inactivation of the PPC impairs fear renewal in a novel context (Figs. 1 and 6); however, these effects induced by inactivation of PPC were not observed in a familiar context (Figs. 2, 3, 4 and 7). Furthermore, the conditioned fear response is a context-independent reaction that occurs before extinction training [2, 4]. Based on our hypothesis, it is unlikely that the PPC mediates the context-independent expression of fear memory and prevents fear retrieval. We also confirmed that the PPC does not affect the retrieval of fear memory (Fig. 5). Therefore, we suggest that the PPC could be a region that processes novel contextual information in the renewal of an extinguished fear memory.

Another possible hypothesis is that the inactivation of PPC may impair recognition of the CS in the novel context. Recently, it is reported that the audition dominates the vision in mice perceiving audio-visual conflicts, and this phenomenon is mediated by PPC [28]. The novel context itself does not evoke contextual fear, but the fear memory to the CS is not erased. Therefore, the conflict may occur between the CS and the novel context. The resolution of this conflict may be mediated by the PPC, and the inactivation of PPC impair this process. Further studies will be required to verify this hypothesis.

It is also important to propose the mechanisms by which the PPC affects neural activity of the amygdala, which is a critical brain region for regulation of the context-dependent fear response [5]. Although no evidence exists of a monosynaptic projection of the PPC to the amygdala, a reciprocal connection to the medial prefrontal cortex (mPFC) has been reported [43–45]. During fear renewal, the activity of the amygdala is modulated by hippocampal and mPFC activity driven by contextual information [6, 11, 46]. In particular, the infralimbic cortex (IL) is reportedly necessary for the suppression of fear after extinction [47–49]. The ventral hippocampal output relieves this suppression, producing a fear relapse through feed-forward inhibition of the IL [13]. Similar to the ventral hippocampus, the PPC possibly conveys input signals to the mPFC. Recent studies may support this idea that parietal-frontal connectivity is enhanced during strong attention [50] and promotes spatial awareness [51]. Further studies will be required to verify the precise functional connections between the PPC and mPFC, and their contribution to fear renewal in a novel context.

In conclusion, the present study reveals the role of the PPC associated with differential modulation in fear renewal, depending on various types of context. Inhibition of the PPC impairs fear renewal in a novel context, but not in a familiar context. These findings may enhance our understanding of the neural mechanism modulating the fear response, depending on the contextual situation.

## Materials and methods

### Experimental subjects

Male C57BL/6 mice, aged 6–10 weeks were selected. Mice were maintained on a 12-h light/dark schedule (lights on at 08:00 am), and food and water were provided ad libitum. All efforts were performed to reduce the number of animals used and to minimize animal suffering.

### Apparatus

A fear conditioning system (Panlab Harvard Apparatus) was used for behavioral tests. The test chamber measuring 250 × 250 × 250 mm, was covered with a sound attenuating box (670 × 530 × 550 mm). Three types of context were used for behavioral experiments. The context A consists of black wall and metallic grid floor, context B comprised a white wall and metallic plate, and context C is made up black and yellow striped paper wall and striped paper floor. The fear conditioning was performed in the context A, and the extinction and fear renewal experiments were conducted in context B and C, respectively. Two types of apparatus were randomly assigned to either context B or C to minimize a biased preference of the mice to specific context.

### Behavioral procedure

On the day of training, mice were acclimated in context A for 5 min, and a CS (2.8 kHz, 85 dB, 30 s) was then co-terminated with an aversive foot shock (US; 0.2 mA, 0.5 s), which was delivered six times for 90-s inter-trial intervals. The extinction sessions were conducted over the next 2 days in context B (extinction context). After exploration for 5 min, the same sound, without an electric foot shock, was presented 12 times a day and intervals were pseudo-randomly assigned at an average of 60 s. On the next day, fear renewal, extinction retrieval and reinstatement tests were performed. The levels of contextual freezing were measured during a pre-CS exploration period (baseline, BL) for 5 min.

For the drug administration studies, the exploration time was 5 min and a single CS was presented. In the fear renewal test, mice were placed in a novel context (context C; Fig. 1) or a familiar context (context A; Fig. 2). Extinction retrieval was conducted in the extinction context (context B; Fig. 3). For fear reinstatement, two electric foot shocks (US; 0.2 mA, 0.5 s) were delivered in context A. After 1 day, freezing behavior was measured to evaluate fear reinstatement in the extinction context (context B; Fig. 4). Retrieval of the conditioned fear memory was tested in a novel context (context C) under conditions in which no extinction took place (Fig. 5).

In the fear renewal test with optogenetics, two CSs were presented and optogenetic inhibition was performed at baseline and during the first CS (Figs. 6 and 7). The level of freezing was scored using the PACKWIN software (Panlab Harvard Apparatus). Freezing was defined as total immobility lasting more than 1 s. The sampling rate was 50 Hz, the software channel gain was 16, and the breathing filter was disabled.

### Stereotaxic surgeries

All surgeries were performed under anaesthesia administered intra-peritoneally, comprising a mixture of ketamine (100 mg/kg) and xylazine (10 mg/kg) in 0.1 M phosphate-buffered saline (PBS). For cannula implantation, a 26-gauge stainless steel guide cannula (Plastics One, C235G-3.4/SPC 1 mm below pedestal) was implanted bilaterally into the PPC (anteroposterior axis [AP] = − 2.0 mm, mediolateral [ML] = ±1.7 mm, dorsoventral [DV] = − 0.5 mm from the bregma) at a 0° angle, and dental cement (Poly-F) was applied to hold the cannula in place and cover the area of the incision. To prevent clogging, a dummy cannula (Plastics One, C235DC/SPC to fit C235G-3.4/SPC 1 mm guide with 0 mm projection) and a dust cap (Plastics One, 303 DC/1) were inserted into the guide cannula.

After surgery, mice were singly housed and allowed to recover for 1 week before behavioral training. For optogenetics experiments, an AAV carrying eNpHR3.0 (AAV2-CamKIIα-eNpHR3.0-eYFP, UNC vector core or AAV5-hSyn-eNpHR3.0-eYFP, Addgene) fused with enhanced yellow fluorescent protein (eYFP) was injected bilaterally into the PPC (AP = − 2.0 mm, ML = ±1.7 mm, DV = − 0.5 mm from the bregma) at a 15° angle. A control virus (AAV2-CamKIIα-eYFP, UNC vector core or AAV5-hSyn-eGFP, Addgene) was injected into the PPC of age-matched mice. The virus was injected at a rate of 0.1 μL/min, and a total of 0.5 μL was administered to each hemisphere using a 5-μL syringe (Hamilton, 7641–01) with a 33-gauge needle (Hamilton, 7762–06). After 2 weeks, mice were again anaesthetised for fiber-optic cannula implantation. A 1-mm fiber-optic cannula (NEWDOON) was bilaterally implanted in the PPC (AP = − 2.0 mm, ML = ±1.7 mm, DV = − 0.5 mm from the bregma) at a 15° angle and dental cement was used to cover the area of the incision. Mice were allowed to recover for 1 week before further behavioral training.

### Drug infusion and optogenetics

To reversibly inactivate the PPC during a behavioral test, optogenetics and the GABA receptor type A agonist, muscimol (Sigma, M1523–5 mg) were both used. Muscimol or a vehicle was injected to the PPC through a 33-gauge stainless steel internal cannula (Plastics One, C235I/SPC to fit a C235G-3.4/SPC 1 mm guide with 0.5 mm projection) connected by a tube to a 5-μL Hamilton syringe (Hamilton, 7634–01), equipped with a syringe pump (Legato 200). A 10 mg/mL stock solution of muscimol was diluted with PBS to 1 mg/mL before infusion. Mice were randomly assigned to either the vehicle (PBS) or the muscimol group. The drug was slowly infused at 0.1 μL/min, and a total of 0.5 μL was infused into each hemisphere. Mice were left for an additional 10 min to allow diffusion of the drug. They were then returned to their respective home cages for 10 min before the behavioral test.

For optogenetics experiments, the implanted fiber-optic cannulae were connected to a fiber-optic patch cord (Doric Lenses, 200 μm core diameter, 0.22 NA) and then connected to a diode-pumped solid-state (DPSS) laser (532 nm, 100 mW). The laser was delivered at 4~5 mW, 50 Hz, and a 50% duty cycle. Light stimulation was administered until the first CS was complete in the fear renewal test.

### Histology

After behavioral experiments, we confirmed placement of cannula tip or viral expression in both hemispheres and excluded the off-target. To verify the intra-PPC placement of the injector cannula tips, mice were perfused with PBS, followed by 4% paraformaldehyde (PFA) solution. The brains were then removed, post-fixed overnight in 4% PFA, and subsequently immersed in 30% sucrose solution at 4 °C. Brain tissues were sectioned (60 μm) on a cryostat (Leica) at − 20 °C. To evaluate the viral infection, brain sections were prepared by the same procedure used for verification of placement of the cannula tip. Staining was performed on free-floating sections.

Brain sections were washed 3 times with PBS for 10 min, and incubated in a blocking solution with 4% normal donkey serum (Jackson ImmunoResearch, 017–000-121) and 0.3% Triton-X in PBS for 1 h. Tissues were then incubated overnight in a blocking solution with primary antibody (1:500, chicken anti-GFP, Aves Labs, GFP-1020) at 4 °C. After being washed three times in PBS for 10 min, tissues were incubated with secondary antibody (1:500, donkey anti-chicken 488, Jackson ImmunoResearch, 703–545-15) in a blocking solution for 2 h at room temperature (20–25 °C). Tissues were again washed in PBS and mounted onto slides with coverslips using the VectaMount permanent mounting medium (Vector). Brain sections were stored in a dark box at 4 °C and imaged using a Pannoramic Scan system (3DHISTECH).

### Statistical analysis

Data were analysed using Prism 8 (GraphPad Software). All data were presented as the mean ± standard error of the mean (SEM). Normality was determined by using the D’Agostino-Pearson normality test. For comparisons of single data points between two groups, the two-tailed unpaired *t-*test was used, while the two-tailed paired *t-*test was conducted for within-subject analysis. For comparisons among data of more than three groups, the two-way repeated measures ANOVA was performed. A *P*-value < 0.05 was considered statistically significant.

## Data Availability

The data supporting the findings of this study are available from the corresponding author upon reasonable request.

## Abbreviations

AAV: Adeno-associated virus
CS: Conditioned stimulus
GABA: Gamma-aminobutyric acid
IL: Infralimbic cortex
NpHR: Halorhodopsin
PPC: Posterior parietal cortex
US: Unconditioned stimulus
YFP: Yellow fluorescent protein
